# Supplementary figures and images for: CRISPR-edited DPSCs constitutively expressing BDNF enhance dentin regeneration in injured teeth
Source: eLife. 2025 Jul 9;14:RP105153. doi: 10.7554/eLife.105153 (PMC12240581; doi:10.7554/eLife.105153)

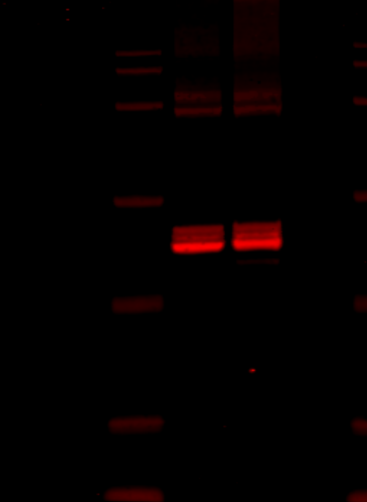

Supplement: Figure 4—source data 1. [file elife-105153-fig4-data1.zip › beta actin.png]

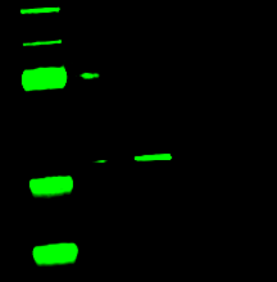

Supplement: Figure 4—source data 1. [file elife-105153-fig4-data1.zip › BDNF over expression.png]

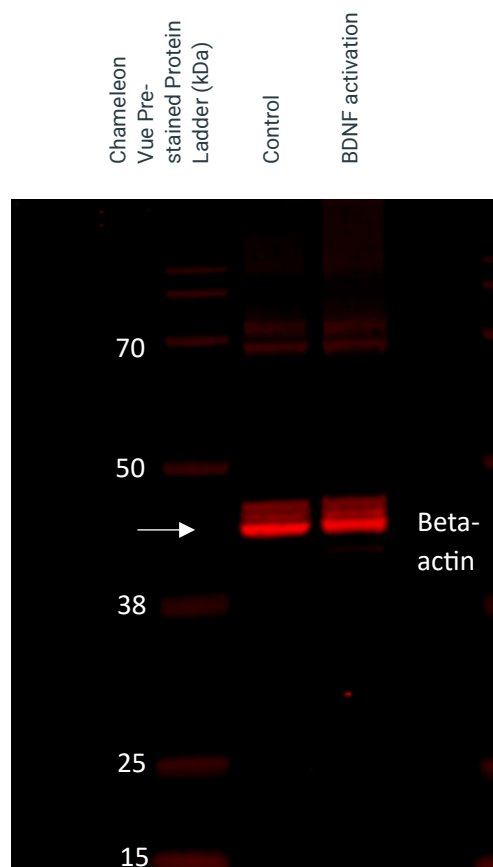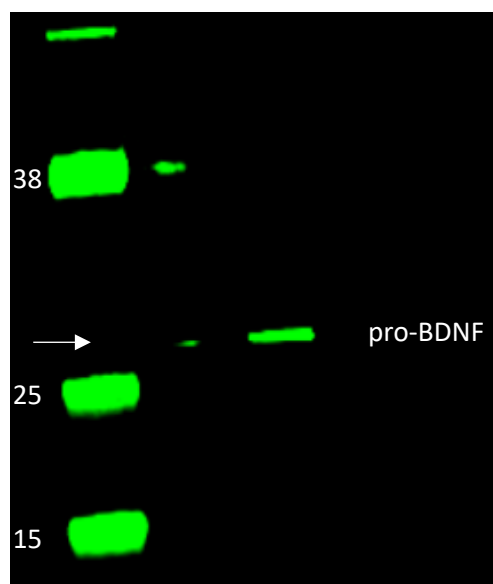

Supplement: Figure 4—source data 2. [file elife-105153-fig4-data2.zip › Original files for western blot images displayed in Fig. 4B.pdf]
